# Plasma proteomics in septic shock and alcohol-related pancreatitis: a hyaluronan-centered approach

**DOI:** 10.1186/s12014-025-09556-2

**Published:** 2025-08-30

**Authors:** Jaap van der Heijden, Asanda Mazubane, Marko Sallisalmi, Egor Vorontsov, Jyrki Tenhunen, Annelie Barrueta Tenhunen

**Affiliations:** 1 Department of Surgical Sciences, Anesthesiology and Intensive Care, Uppsala University, 75185 Uppsala, Sweden; 2 Division of Anaesthesia and Intensive Care Medicine, Intensive Care Units, Department of Surgery, Helsinki University Hospital, 260 Helsinki, Finland; 3 Proteomics Core Facility, Sahlgrenska Academy, University of Gothenburg, 41390 Gothenburg, Sweden

**Keywords:** Proteomics, Sepsis, Pancreatitis, Hyaluronan, Hyaluronidase, Hyaluronidase inhibition, Inter-alpha-trypsin inhibitor, Extracellular matrix, Glycocalyx

## Abstract

**Background:**

Sepsis is a critical condition characterized by a dysregulated immune response to infection. As sepsis develops to septic shock, its most severe form, morbidity and mortality increases. Hyaluronan is a key component of the extracellular matrix and the endothelial glycocalyx. In sepsis, plasma hyaluronan concentrations are increased and correlate with disease severity. In this study we aimed to explore and compare the proteomic profiles of hyaluronan-associated proteins in patients with the dysregulated immune response of septic shock and the sterile inflammation of acute alcohol-related pancreatitis.

**Methods:**

The present study involved proteomic analysis of patients with septic shock (n = 13), pancreatitis (n = 8), and healthy controls (n = 8). LC–MS/MS was conducted for peptide analysis. Hyaluronan-associated proteins were identified using the UniProt REST API, followed by functional and pathway enrichment analyses with GOATOOLS and GSEApy. Statistical analyses, including ANOVA and post hoc tests, were performed using Python and SPSS, with significance set at p < 0.05.

**Results:**

From a total sum of 663 detected unique plasma proteins, 15 were identified as hyaluronan-related proteins. Plasma levels of 11/15 proteins separated septic shock from pancreatitis in a statistically significant manner. Between the groups differences were apparent on day 1 (8 proteins in septic shock versus 3 in pancreatitis) and day 4 (6 proteins in septic shock versus 3 in pancreatitis) relative to controls. Functional enrichment analysis revealed associations with extracellular matrix organization, proteolytic enzyme regulation, and hyaluronan metabolism. Notably, members of the inter-alpha-inhibitor family demonstrated distinct patterns, with ITIH3 levels increasing and ITIH1, ITIH2, and ITIH4 levels decreasing in septic shock compared to controls. Additionally, plasma hyaluronidase inhibition correlated positively with ITIH3 levels.

**Conclusion:**

The present study explored the role of hyaluronan-related proteins in septic shock pathophysiology, revealing potential dysregulation associated with sepsis severity. The decrease in ITIH1, ITIH2 and ITIH4, as compared to the increase in ITIH3, suggest a complex alteration in the protein balance of the IαI-family in sepsis. Overall, the altered proteomic profile of hyaluronan-related proteins as reflected by the GO terms indicates a complex dysregulation not only in hyaluronan metabolism and extracellular matrix, but also in the regulation of several proteolytic enzymes. Future studies on this area are warranted.

**Supplementary Information:**

The online version contains supplementary material available at 10.1186/s12014-025-09556-2.

## Background

Sepsis is a global health concern with high morbidity and mortality [1]. The complexity of the sepsis syndrome is contained in its definition as a dysregulated immune response to infection [2]. Different phenotypes and endotypes have been described [3–5] and further clarified through several proteomics studies delineating differentially expressed proteins in sepsis; identifying biomarkers and fundamental molecular mechanisms [6–11].

Hyaluronan, a phylogenetically well-conserved glycosaminoglycan (GAG), particularly abundant in connective tissue [12], is an important component of the extracellular matrix (ECM) and endothelial glycocalyx layer [13]. High molecular weight hyaluronan (> 1000 kDa) exhibits anti-inflammatory characteristics and is the dominating molecular weight fraction in vivo [14, 15]. Degraded hyaluronan [16–18], on the contrary, is a signal of tissue damage and promotes inflammation [19].

Concentrations of hyaluronan are elevated in sepsis and correlate with disease severity [20, 21]. While this raise in serum concentration of hyaluronan decreases during the 1 st week in sepsis-survivors, the same decline is not detected in non-survivors [22]. Further, acute kidney injury and mortality in sepsis and ARDS is predicted by urinary excretion of hyaluronan [23]. Hyaluronan increase is also positively associated with cumulative fluid volumes and organ failure [24] and crystalloid infusion results in plasma hyaluronan increase [25], even in independence of renal clearance [26]. However, hyaluronan turn-over in plasma is fast [12] and its role and metabolism not fully understood in sepsis pathophysiology.

We previously showed that the sepsis associated increase in plasma hyaluronan levels correspond with a decrease in effective plasma hyaluronidase activity, as well as an increase in endogenous plasma hyaluronidase inhibition. However, in acute pancreatitis the decrease in plasma hyaluronidase activity was not associated with increased plasma hyaluronan concentrations or hyaluronidase inhibition [27]. Little is known about other hyaluronan-related proteins and their potential effects in septic shock and pancreatitis. To address this gap of knowledge, we explored, with a hyaluronan-centered approach, plasma proteomics in healthy volunteers and patients with septic shock and compared plasma proteomics profiles between septic shock (dysregulated immune response to infection) and acute pancreatitis (sterile inflammation).

## Methods

### Participants and sample handling

The current study is a subgroup analysis from previous published material [28]. Patients with septic shock (n = 13) and patients with pancreatitis (n = 8) who were admitted to the intensive care unit at the Helsinki University Hospital were enrolled as consecutive patients from September 2008 to September 2009. Healthy volunteers (n = 8) were included as controls. Inclusion criteria for patients with septic shock were: age ≥ 18 years, ≥ 2 systemic inflammatory response syndrome (SIRS) criteria fulfilled, norepinephrine therapy ≥ 0.1 mcg/kg/min despite of adequate fluid resuscitation to maintain systemic arterial pressure above 90 mmHg, and highly suspected infection or confirmed positive blood cultures. The inclusion criteria for patients with pancreatitis were: age ≥ 18 years, acute pancreatitis confirmed by computer tomography scan and whose etiology was excessive use of ethyl alcohol, ≥ 2 of the SIRS criteria fulfilled and the need of an organ support therapy (i.e. need for vasopressor, renal replacement therapy or mechanical ventilation). Whole blood was collected in K2-EDTA-tubes within 24 h of admission to the intensive care unit (day 1) and 72 h thereafter (day 4). The samples were centrifuged at 18 °C for 15 min at 2500*g* and the plasma was aspirated and stored in aliquots at − 80 °C until further analysis.

### Sample preparation for proteomic analysis

Proteomic analysis was conducted in 2017 and 2018, utilizing samples that were thawed for the first-time following storage. The samples were processed in a blinded manner to ensure objectivity in the analysis. Abundant plasma proteins were depleted by treating individual samples with Pierce™ Top 12 Abundant Protein Depletion Spin Columns (Thermo Fisher Scientifc; PN 85165). For each sample, the filtrate was spiked with sodium dodecyl sulfate (SDS) to 2% final concentration, triethylammonium bicarbonate (TEAB) to 50 mM and DL-dithiothreitol (DTT) to 100 mM final concentration. Samples were incubated at 60 °C for 30 min, diluted to 1:4 by 8 M urea solution and transferred onto Nanosep 30 k Omega filters (Pall Corporation, Port Washington, NY, USA) to be processed via the modified filter-aided sample preparation (FASP) method (Wisniewski JR et. al. Nat Methods. 2009 May;6(5):359–62). In brief, samples were washed 4 times by 8 M urea, and free cysteine residues were modified by 10 mM methyl methanethiosulfonate (MMTS) solution in the digestion buffer (0.5% sodium deoxycholate (SDC), 50 mM TEAB) for 30 min at room temperature, and the filters were then repeatedly washed with 100 µl of digestion buffer. Pierce trypsin protease (MS Grade, Thermo Fisher Scientific) in digestion buffer was added at a ratio of 1:100 relative to total protein mass and the samples were incubated at 37 °C overnight; another portion of trypsin (1:100) was added and the mixture was incubated at 37 °C for 3 h.

Individual peptide samples were collected by centrifugation and labelled using Tandem Mass Tag (TMT 10plex) reagents (Thermo Fischer Scientific) according to the manufacturer’s instructions. Labelled samples were combined into pooled 10plex samples, concentrated using vacuum centrifugation, and SDC was removed by acidification with 10% trifluoroacetic acid (TFA) and subsequent centrifugation. The pooled samples were fractionated using an ÄKTA (GE Healtcare) or a Dionex Ultimate 3000 UPLC system (Thermo Fischer Scientific). Peptides were separated into 40–44 primary fractions by basic reversed-phase chromatography (bRP-LC) on an XBridge BEH C18 column (3.5 μm, 3.0 × 150 mm, Waters Corporation) using 10 mM ammonium formate buffer at pH 10.00 as solvent A and 90% acetonitrile, 10% 10 mM ammonium formate at pH 10.00 as solvent B. The primary fractions were concatenated into final 20 fractions, dried and reconstituted in 3% acetonitrile, 0.2% formic acid for LC–MS analysis.

### LC–MS/MS analysis

Each final fraction was analyzed on an Orbitrap Fusion Tribrid or Q Exactive HF mass spectrometer interfaced with an Easy-nLC 1000 or 1200 liquid chromatography system (all-Thermo Fisher Scientific). Peptides were trapped on an Acclaim Pepmap 100 C18 trap column (100 μm × 2 cm, particle size 5 μm, Thermo Fischer Scientific) and separated on an analytical column (75 μm × 35 cm, packed in-house with Reprosil-Pur C18, particle size 3 μm, Dr. Maisch, Ammerbuch, Germany) at a flow of 300 nL/min using 0.2% formic acid in water as solvent A and 80% acetonitrile, 0.2% formic acid as solvent B. Peptides were separated over a 60 min-long gradient elution method. On the Orbitrap Fusion, MS scans were performed at 120,000 resolution. The most abundant doubly or multiply charged precursors from the MS1 scans were isolated using the quadrupole with 0.7 m/z isolation window with a “top speed” duty cycle of 3 s and dynamic exclusion enabled. The isolated precursors were fragmented by collision induced dissociation (CID) and detected in the ion trap, followed by multinotch (simultaneous) isolation of the top fragment ions ions, fragmentation (MS3) by higher-energy collision dissociation (HCD) and detection in the Orbitrap. On the Q Exactive HF, most abundant precursor ions were selected from MS1 spectra and fragmented using HCD with dynamic exclusion enabled.

### Proteomic data analysis

Identification and relative quantification was performed using Proteome Discoverer version 2.4 (Thermo Fisher Scientific). The database search was performed using the Mascot search engine v. 2.5.1 (Matrix Science, London, UK) against the Swiss-Prot Homo sapiens database. Trypsin was used as a cleavage rule with no missed cleavages allowed; methylthiolation on cysteine residues, TMT 6plex at peptide N-termini and on lysine side chains were set as static modifications, and oxidation on methionine was set as a dynamic modification. For the total proteome analysis, precursor mass tolerance was set at 5 ppm and fragment ion tolerance at 0.02 Da (Q Exactive HF) and 0.6 Da (Fusion). Percolator was used for PSM validation with the strict FDR threshold of 1% in both cases.

Quantification was performed in Proteome Discoverer 2.4. TMT reporter ions were identified with 3 mmu mass tolerance in the MS2 (Q Exactive HF) or MS3 (Fusion) HCD spectra, and the TMT reporter S/N values for each sample were normalized within Proteome Discoverer 2.4 on the total peptide amount. Only the unique identified peptides were taken into account for the protein quantification.

### Identification and selection of hyaluronan-associated genes

The identification of hyaluronan-associated genes was performed using Python 3.10.12. The UniProt REST Web Application Programming Interface (API) was used to query and retrieve gene annotations that met specific criteria (UniProt Consortium). A keyword-based query, utilizing the terms “hyaluronan,” “hyaluronic acid,” “hyaluronidase,” “hyaluronic acid synthase,” “hyaluronate binding protein,” “hyaluronan oligosaccharides,” “hyaluronan synthase,” and “hyaluronan receptor,” was executed across our dataset of 663 genes. Gene symbols were returned as “hits” when the query keywords were found in the annotations, including functional comments, Gene Ontology (GO) terms, and cross-referenced databases, formatted in JSON.

### Functional enrichment analysis

Functional enrichment analysis was conducted on the identified hyaluronan-associated proteins using Python with GOATOOLS v1.4.12 [29]. To ensure the specificity of the enriched terms, the analysis was performed without propagating up to parent terms. A p-value cutoff of ≤ 0.05 was set as the threshold for statistical significance, and the Benjamini–Hochberg procedure was applied for controlling the false discovery rate. The significantly enriched Gene Ontology (GO) terms were compiled, and the results were saved for further interpretation and visualization. Subsequently, Python was employed to generate a bar plot of the top significantly enriched GO terms using the matplotlib v3.9.2 library [30].

### Pathway enrichment analysis

Pathway enrichment analysis was conducted using Python with GSEApy v1.1.3 [31]. The enrichr method was employed to compare the identified hyaluronan-associated proteins against the “Reactome_2022” pathway database for humans, aiming to identify pathways significantly enriched with genes from the list. An adjusted p-value cutoff of ≤ 0.05 was set as the threshold for statistical significance. The top 20 significantly enriched pathways were compiled, and the results were saved for further interpretation and visualization. Subsequently, Python was utilized to generate a dot plot of these top 20 significantly enriched pathways using the matplotlib v3.9.2 library [30].

### Plasma hyaluronidase inhibition assay

Quantification of endogenous plasma hyaluronidase inhibition in the patients is described in detail in our previous publication [27]. In summary, 96-well microtiter plates (Covalink, Nunc) coated with Sulfo-NHS (0.184 mg/ml), biotin-labeled HA (0.2 mg/ml) and EDAC (0.123 mg/ml) were stored overnight at 4 °C. Plasma samples (900× dilution, containing 1 TRU/ml bovine testicular hyaluronidase) and the standard curve (1 to 1 × 10–4 TRU/well) were added to individual wells in duplicates and incubated for 60 min. After incubation with streptavidin-HRP and TMB, the final reaction was terminated with 2 M H2SO4 and absorbance was measured at 450 nm. The plasma hyaluronidase inhibition was calculated in percent as follow: inhibition (%) = 1 − ((Amax − Asample)/(Amax − Amin)) where Amax, Amin and Asample were the absorbance of the wells of the positive control, the wells exposed to only bovine hyaluronidase (no inhibitor) and the samples containing both bovine hyaluronidase and plasma inhibitor respectively.

### Statistics for patient related data and selected proteins

The protein-level tables of the proteomic data were exported and loaded into Python 3.12.0 for merging and statistical processing using ANOVA including Benjamini–Hochberg procedure for multiple testing, and post hoc Tukey’s HSD test. Analysis for patients’ characteristics, clinical and laboratory data as well as visualizing the boxplots of the hyaluronan-related proteins and correlations were done with IBM® SPSS® statistics version 23 (SPSS, Inc., Chicago, IL, USA). Categorial data was tested with the Chi-square test. Numerical data was tested with the shapiro wilk test for normality. Independent data between the groups was analyzed with the Kruskal–Wallis or Mann–Whitney U when appropriate. Dependent data between day 1 and day 4 was analyzed with the Wilcoxon test. Based on the finding of some of the hyaluronan-related proteins, correlations with hyaluronidase from our previous study [27] was performed with the Spearman’s rank-order test as the statistical method. The significance level was set at < 0.05 and data was reported as median and interquartile range (IQR) or percentage (%).

## Results

### Patients characteristics, clinical data

There was no difference in age, sex or height between the three groups. Patients, both with septic shock and pancreatitis had higher weight compared with controls (p < 0.05 and < 0.01 respectively). SOFA score was higher in patients with septic shock as compared with pancreatitis on day 1 (p < 0.01) and day 4 (p < 0.05). In patients with septic shock the mean arterial pressure increased from day 1 to day 4 (p < 0.01) while noradrenalin infusion rates decreased (p < 0.05). This was not seen in patients with pancreatitis. Blood cultures were positive in 8 of 13 (61.5%) patients with septic shock and *Streptococcus pneumoniae* (n = 4) was the most common finding. For detailed information about patients’ characteristics, clinical and microbiology data see supplemental table S1, S2 and S3.

### Hyaluronan-associated proteins

**Fig. 1:**
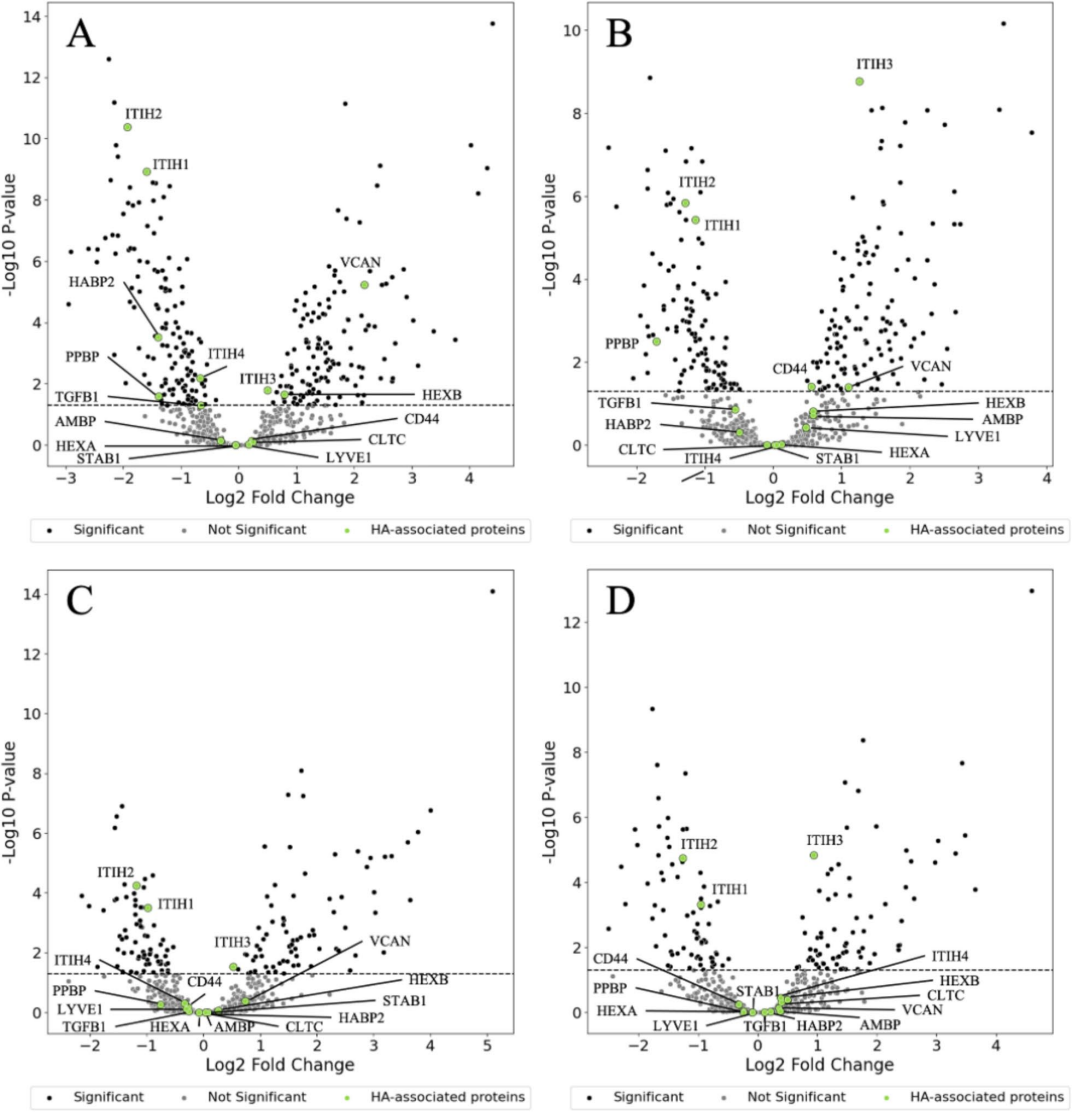
Volcanoplots for controls versus patients with septic shock on day 1 (**A**) and day 4 (**B**) and controls versus patients with pancreatitis on day 1 (**C**) and day 4 (**D**). A total of 663 proteins are visualised as single dots in each volcanoplot. The y-axis represents -log10 transformed p-values and the x-axis represents the log2 fold change. Black and gray dots represent statistically significant (p < 0.05) and non-significant proteins, respectively. Green dots represent the 15 hyaluronan-related proteins

**Fig. 2:**
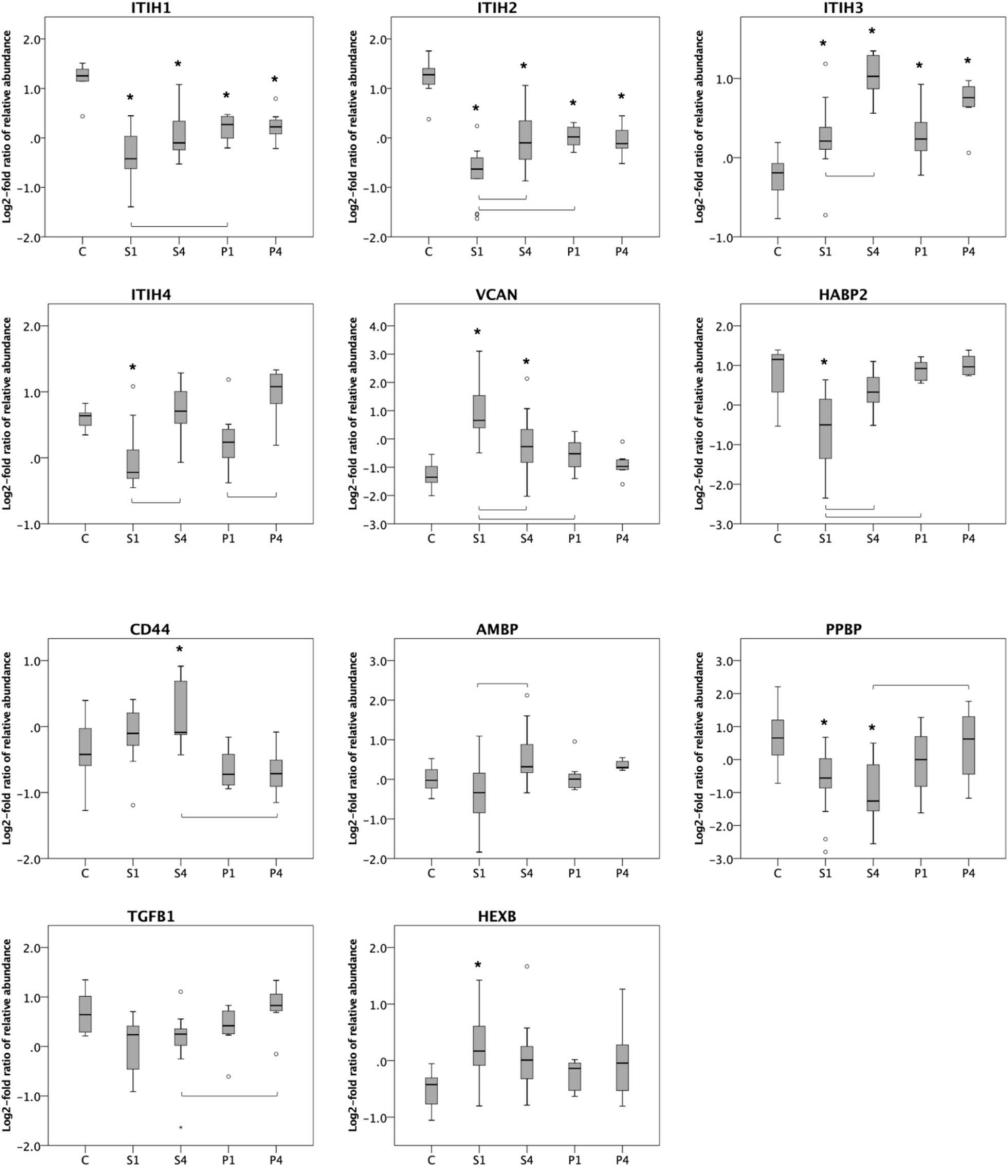

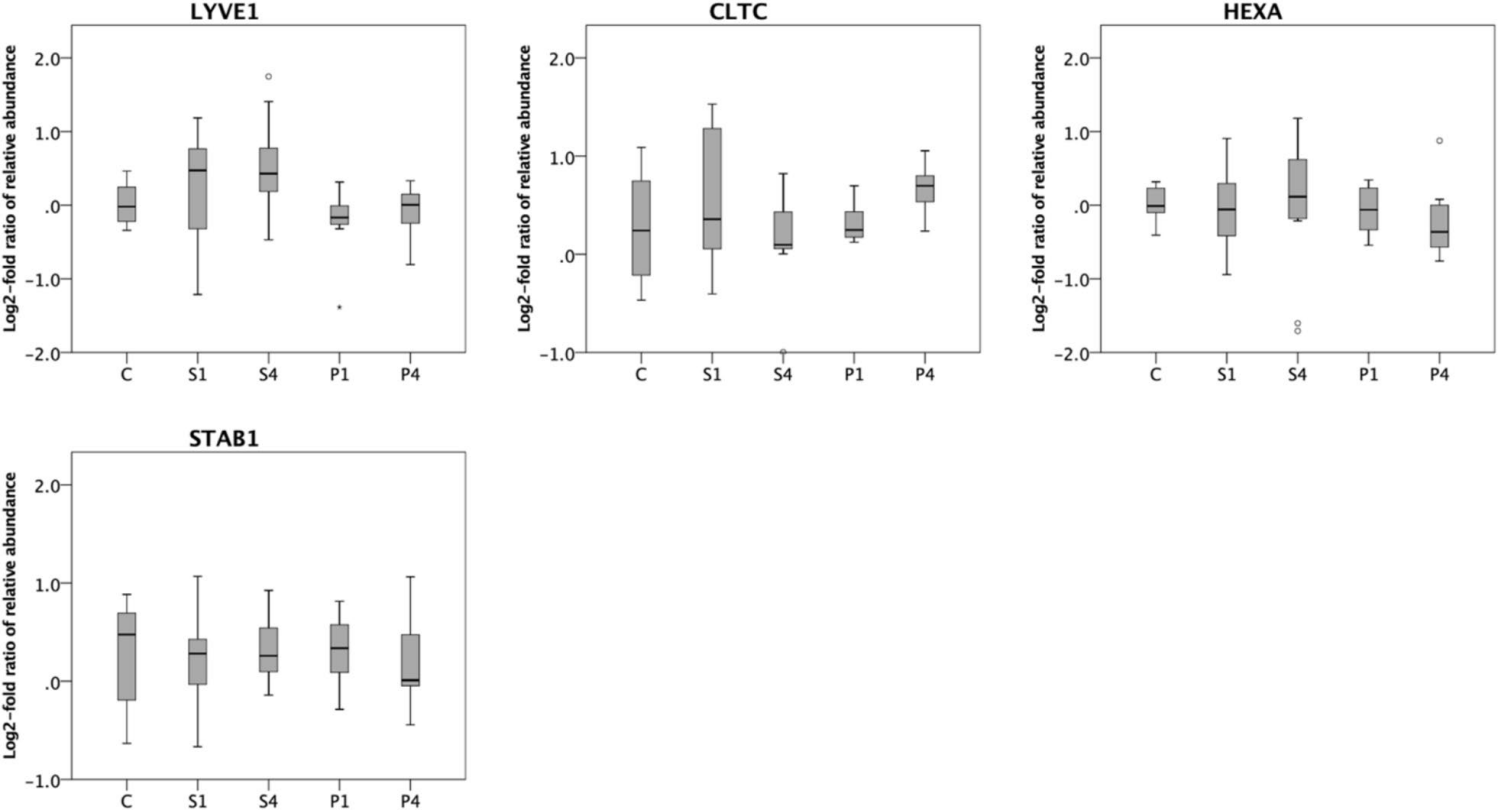
Hyaluronan-related proteins for controls (C), patients with septic shock on day 1 (S1) and day 4 (S4) and patients with pancreatitis on day 1 (P1) and day 4 (P4). ANOVA was performed including Benjamini–Hochberg procedure. LYVE1, CLTC, HEXA and STAB1 were not significant (n.s.). Post hoc analysis with Tukey’s HSD test was performed for the remaining 11 proteins. * significant compared with control (p < 0.05), brackets show significance between day 1 and day 4 for patients with septic shock and pancreatitis (p < 0.05)

**Fig. 3:**
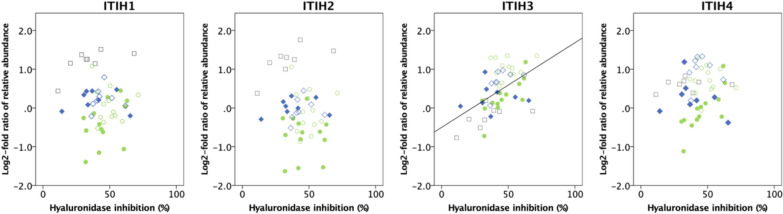
Spearman’s rank-order correlation between log2-fold ratio of relative abundance of ITIH 1–4 and endogenous hyaluronidase inhibition in plasma. Gray/squares represent controls, green/circles represent patients with septic shock on day 1 (filled) and day 4 (non-filled), blue/diamonds represent patients with pancreatitis on day 1 (filled) and day 4 (non-filled). A correlation between ITIH3 and plasma hyaluronidase inhibition was found (rs = 0.517, p < 0.001)

A total of 663 unique plasma proteins were found in plasma of controls and patients with septic shock and pancreatitis. The top-10 most upregulated and downregulated proteins in patients with septic shock and pancreatitis, compared to controls at day 1, are presented in supplementary tables S4 and S5, respectively. Using the keywords as stated in the methods we identified 15 hyaluronan-associated proteins (Fig. 1). Plasma levels of four proteins did not differ between the groups (LYV1, CLTC, HEXA and STAB1, p > 0.05). Of the remaining 11 hyaluronan-related proteins, eight proteins were significantly up- or downregulated in patients with septic shock compared with controls on day 1 and six proteins on day 4. Three proteins were significant up- or downregulated in pancreatitis on day 1 and on day 4. Six of the hyaluronan-related proteins changed significant in patients with septic shock between day 1 and 4 compared with one protein in patients with pancreatitis. Four of the hyaluronan-related proteins differed significant between patients with septic shock and pancreatitis on day 1 while three proteins differed between the two conditions on day 4 (Fig. 2). A positive correlation between endogenous plasma hyaluronidase inhibition from our previous study [27] and ITIH3 but not ITIH1, ITIH2 or ITIH4 was found (Fig. 3). For sub-analysis of correlations between ITIH 1–4 and endogenous plasma hyaluronidase inhibition for control, patients with septic shock (day 1) and pancreatitis (day 1) see supplemental table S6.

### Gene Ontology and pathway enrichment analysis

**Fig. 4:**
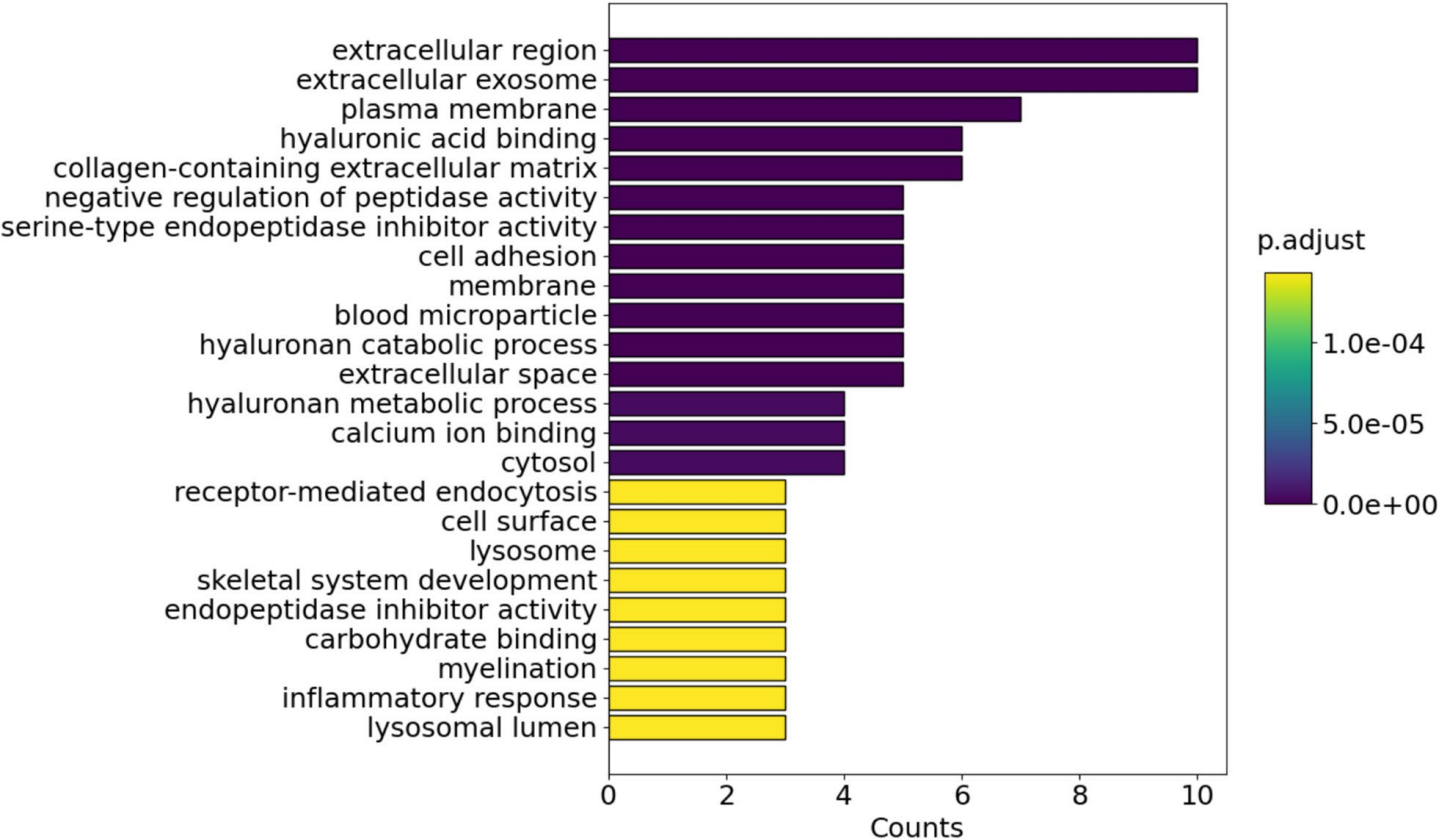
Bar plot illustrating the results of the functional enrichment analysis of the 15 identified hyaluronan-associated proteins. A total of 24 enriched Gene Ontology (GO) terms with a minimum protein count of three are presented. The y-axis represents the GO terms related to biological processes, metabolic functions, or cellular components, while the x-axis represents the counts, indicating the number of hyaluronan-associated proteins enriched in each term. The color gradient from purple to yellow indicates adjusted p-values (p < 0.05) corresponding to each bar, where darker shades (purple) signify lower p-values and lighter shades (yellow) indicate higher p-values

**Fig. 5:**
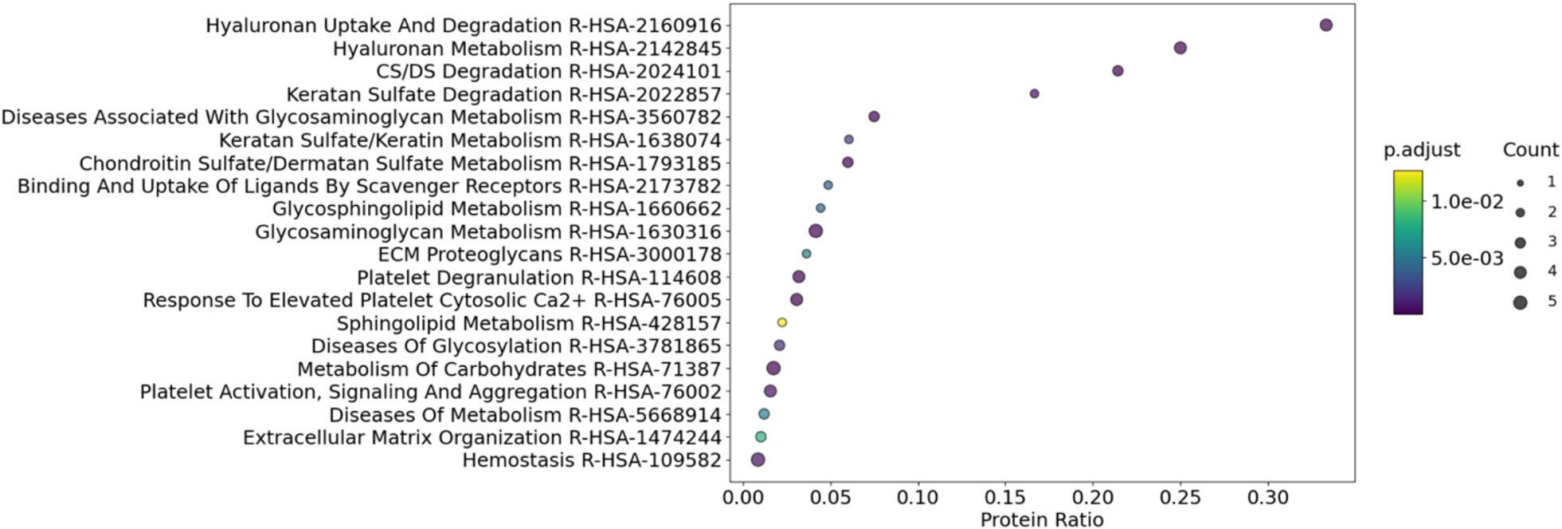
Dot plot illustrating the Reactome pathway enrichment analysis of the 15 identified hyaluronan-associated proteins. The top 20 significantly enriched pathways are presented. The y-axis lists the pathways along with their corresponding Reactome IDs, and the x-axis represents the protein ratio, calculated as the fraction of hyaluronan-associated proteins overlapping with the total protein count of each pathway. The color gradient from purple to yellow indicates adjusted p-values (p < 0.05) corresponding to the dot colors, where darker shades (purple) signify lower p-values and lighter shades (yellow) indicate higher p-values. The size of the dots reflects the count of hyaluronan-associated proteins involved in each pathway, with larger dots representing pathways with a higher number of associated proteins

Analysis of the distribution of 15 hyaluronan-related proteins across the GO database resulted in a total of 24 GO terms with a minimal protein count 3 per term and p < 0.05 (Fig. 4). A complete list of GO terms with their corresponding protein counts is provided in the supplementary material (Supplemental table S7). Pathway enrichment analysis comparing the 15 hyaluronan-related proteins against the “Reactome_2022’ pathway database for humans was limited to the 20 most significant findings (Fig. 5).

## Discussion

In the present study, we applied a hyaluronan-focused approach in proteomics to investigate hyaluronan-related proteins in patients with septic shock and pancreatitis. Our proteomic analyses identified 663 unique proteins, from which we performed a targeted search for hyaluronan-related proteins. This approach allowed us to identify 15 hyaluronan-related proteins and compare their plasma levels among patients with septic shock, pancreatitis and controls at day 1 and day 4 after admission to the intensive care unit.

Among the 15 proteins, four showed no significant differences between the groups. Plasma levels of 11/15 proteins separated septic shock from pancreatitis in a statistically significant manner, with differences observed on day 1 (8 versus 3) and day 4 (6 versus 3), when compared to the controls. Additionally, the comparison between day 1 and day 4 revealed a more pronounced difference in plasma levels in patients with septic shock than in those with pancreatitis (6 versus 1).

The hyaluronan-related proteins and their relation to the extracellular matrix organisation is reflected by several associated Gene Ontology (GO) terms, including ‘extracellular exosomes’ (GO:0070062), ‘plasma membrane’ (GO:0005886), and ‘collagen-containing extracellular matrix’ (GO:0062023) as well as the pathway ‘extracellular matrix proteoglycans’ (R-HSA-3000178). The GO terms ‘hyaluronic acid binding’ (GO:0005540), ‘hyaluronan catabolic process’ (GO:0030214) and ‘receptor-mediated endocytosis’ (GO:0006898) as well as the pathways ‘hyaluronan metabolism’ (R-HSA-2142845) and ‘hyaluronan uptake and degradation’ (R-HSA-2160916) confirm the role of some of the hyaluronan-related proteins in the metabolism and utilisation of hyaluronan. Lastly, the GO terms ‘serine-type endopeptidase inhibitor activity’ (GO:0004867) and ‘negative regulation of peptidase activity’ (GO:0010466) indicate a role of hyaluronan and related proteins in the regulation and inhibition of several proteolytic enzymes. In the following, we provide a detailed description of some of these processes and their relevance to our findings.

### Regulation and inhibition of proteolytic enzymes: the inter-alpha-inhibitor family

Five of the 15 hyaluronan-related proteins, specifically ITIH 1–4 and AMBP, are essential components of the inter-alpha-inhibitor family (IαI-family). This family consist of proteins characterised by serine-type endopeptidase inhibitor. The key components for the IαI-family include the light chain Bikunin and 6 known inter-alpha-trypsin inhibitor heavy chains isoforms (ITIH 1–6) [32]. While free Bikunin is renowned for inhibiting a wide range of proteases [33], other IαI-family proteins, particularly ITIH 1–3, interact with hyaluronan to stabilise the extracellular matrix, forming covalent bonds [34, 35]. The ITIHs are also involved in the inflammatory response, either in conjunction with Bikunin or independently [33].

The synthesis of the IαI-family begins with the precursor AMBP, which is proteolytically cleaved into Bikunin. Bikunin contains a chondroitin sulphate chain that can covalently link with ITIH. The binding of a single ITIH3 to Bikunin forms the pre-alpha-inhibitor (PαI) protein, while the binding of ITIH1 and ITIH2 together to Bikunin results in the formation of the inter-alpha-trypsin inhibitor protein (IαI-protein). In plasma, Bikunin can further exist as a free unbound protein or as a complex bound to a single ITIH1, ITIH2 or ITIH5 [36]. In contrast, ITIH4 lacks the possibility to bind Bikunin and is exclusively found as a free isoform in plasma [32].

The total IαI-family protein levels are lower in patients with septic shock compared with controls [37], and failure of recovery of plasma levels is associated with higher mortality [38]. Although we did not measure total IαI-family protein levels, our findings indicate that plasma levels of all ITIHs, except for ITIH3, were decreased in patients with septic shock on day 1. Furthermore, all ITIHs showed signs of recovery between day 1 and day 4 in patients with septic shock, consistent with previous reports. There are studies that indicate that treatment with free Bikunin reduce mortality in patients with sepsis [39, 40] and improve outcome in patients with severe acute pancreatitis [41].

The role of different ITIHs in the context of sepsis is not fully elucidated. Patients with bacterial infection had lower plasma levels of AMBP and ITIH2, an increase in ITIH3, while ITIH1 remained unchanged compared with healthy controls [42]. Our study supports the findings regarding ITIH2 and ITIH3, but we observed a significant decrease in ITIH1 plasma levels in patients with septic shock while AMBP remained unchanged. This discrepancy may reflect the more severe illness of patients in our cohort (septic shock versus bacterial infection), leading to lower ITIH1 levels. Yet another study found increased ITIH3 levels in septic versus non-septic intensive care patients [43], which aligns with our results. Additionally, our study indicates elevated ITIH3 plasma levels in patients with pancreatitis. The increased ITIH3 plasma levels in patients with septic shock and pancreatitis suggest that changes in ITIH3 are inflammation-dependent rather than directly driven by infection.

The dynamics of ITIH4 levels in sepsis and septic shock are not conclusive. One study found no significant difference in ITIH4 levels between patients with septic shock and healthy controls over the first three consecutive days following admission to the intensive care unit [44]. In contrast, another study demonstrated a significant decrease in ITIH4 levels in patients with sepsis upon hospital admission compared to healthy controls, which aligns with our findings. Notably, the inability to recover ITIH4 levels is associated with a higher mortality [45].

The clinical significance of changes in IαI-family plasma levels during sepsis and pancreatitis is currently unknown. As noted earlier, IαI-family members, including Bikunin, inhibits several proteases that are elevated in both conditions, such as leukocyte elastase, cathepsin G, trypsin, plasmin, and kallikrein. Regarding hyaluronan, the IαI-family members and in particular PαI (i.e. Bikunin bound to ITIH3), are believed to be the primary inhibitors of plasma hyaluronidase [46]. In the current study, ITIH3 plasma levels were increased in patients with septic shock and in those with pancreatitis. Our previous research showed that plasma hyaluronidase inhibition increases in patients with septic shock, while no increase was observed in those with pancreatitis [27]. Therefore, changes in ITIH3 levels alone do not fully explain plasma hyaluronidase inhibition in septic shock and pancreatitis but may, nevertheless, contribute. Additionally, the increase in plasma hyaluronidase inhibition correlates with the ITIH3 plasma levels found in this study, further supporting the findings by Mio et al. [46]

### Extracellular matrix and glycocalyx

GO terms and pathway enrichment analyses illustrate the role of hyaluronan-related proteins in the ECM. The organization of these components is complex, including GAGs, proteoglycans, glycoproteins, and various transmembrane receptors. Together, they are essential for tissue integrity, cell adhesion, migration, and homeostasis. Disruption of the ECM, including the glycocalyx, in conditions such as sepsis and pancreatitis plays a role in the dysregulated state of inflammation and coagulation, resulting in vascular leakage and edema [47].

Hyaluronan is the longest GAG and serves as a major component of both the ECM and glycocalyx. The transmembrane receptor CD44, which specifically binds hyaluronan, is the most abundant receptor of its kind. During inflammation, CD44 expression increases on leukocytes, and the CD44-hyaluronan interactions are crucial for recruiting and activating immune cells. Elevated plasma levels of CD44 have been observed in patients with septic shock and are associated with impaired microvascular function [48]. Our study found increased soluble CD44 plasma levels in patients with septic shock four days after admission, while no change was noted in patients with pancreatitis.

The LYVE1 receptor shares structural similarities with CD44 but is predominantly localized in the lymphatic system. Most of the hyaluronan produced in the body is transported through lymphatic vessels and degraded in lymph nodes before entering the circulation [49], and LYVE1 facilitates this transport. In contrast to the CD44-hyaluronan interaction that promotes leukocyte exit from the bloodstream, the LYVE1-hyaluronan interaction aids in the docking, entry, and migration of leukocytes into lymphatic vessels [50]. Limited information is available about LYVE1 in septic shock; however, our study identified plasma soluble LYVE1 levels at comparable levels in healthy volunteers, septic shock and pancreatitis.

Versican, a proteoglycan encoded by the VCAN gene, is another significant protein in the ECM that interacts with hyaluronan. Its levels increase alongside hyaluronan during early inflammation, contributing to the inflammatory response [51]. In our study, plasma versican levels increased in patients with septic shock on day 1, compared to both controls and pancreatitis. No increase was detected in patients with pancreatitis compared to controls. As seen with other extracellular components, cleavage of versican results in smaller fragments that exhibit both pro-inflammatory and anti-inflammatory properties. Versican may also play a role in the formation of serum-derived hyaluronan-associated proteins (SHAPs) [52].

Another glycoprotein of interest in the ECM is HABP2. High molecular weight hyaluronan can bind to and inhibit the serine protease activity of HABP2. Conversely, low molecular weight hyaluronan activates HABP2, inducing activation of protease-activated receptors (PARs), which leads to various intracellular responses such as disruption of the endothelial cell barrier [53]. Due to its multifunctional nature, HABP2 is also referred to as factor VII-activating protease (FSAP), reflecting its role in activating factor VII within the coagulation cascade. Our study found a decrease in HABP2 plasma levels in patients with septic shock on day 1, but not on day 4. There were no changes in HABP2 levels in patients with pancreatitis compared to controls.

### Limitations

The present study has several limitations. Most importantly the findings of the present study are the result of post-hoc analyses. Even though we formulated the hypothesis before we performed the search of hyaluronan related proteins, the study sampling was not designed to specifically address the study question. Therefore, the results can only be hypothesis generating, requiring further confirmation in future studies. Another important limitation is that the study is based on a relatively small sample size, consequently, small variations between groups might not have been detected.

## Conclusion

In the present study we identified 15 hyaluronan-related proteins, of which 11 differed in expression between septic shock and pancreatitis. The decreased plasma levels of ITIH1, ITIH2 and ITIH4, as compared to the increased levels of ITIH3, suggest a complex alteration in the protein balance of the IαI-family in the pathophysiology of sepsis. Furthermore, the increase in plasma concentrations of ITIH3 with its corresponding rise of plasma hyaluronidase inhibition in patients with septic shock suggest that PαI might be the primary inhibitor of hyaluronidase in septic shock. Overall the altered proteomic profile of hyaluronan-related proteins as reflected by the GO terms indicates a complex dysregulation not only in hyaluronan metabolism and extracellular matrix, but also in the regulation of several proteolytic enzymes in septic shock and to some extent in pancreatitis.

## Supplementary Information

Supplementary Material 1

## Data Availability

No datasets were generated or analysed during the current study.

## Abbreviations

ANOVA: Analysis of variance
AMBP (hyaluronan-related proteins): Protein AMBP
API: Application programming interface
CD44 (hyaluronan-related proteins): CD44 Antigen
CID: Collision induced dissociation
CLTC (hyaluronan-related proteins): Clathrin heavy chain 1
DTT: DL-Dithiothreitol
ECM: Extracellular matrix
FASP: Filter-aided sample preparation
FSAP: Factor VII-activating protease
GAG: Glycosaminoglycan
GO: Gene ontology
HABP2 (hyaluronan-related proteins): Hyaluronan-binding protein 2
HCD: Higher-energy collision dissociation
HEXA (hyaluronan-related proteins): Beta-hexosaminidase subunit alpha
HEXB (hyaluronan-related proteins): Beta-hexosaminidase subunit beta
IαI-family: Inter-alpha-inhibitor family
IαI-protein: Inter-alpha-trypsin inhibitor protein (Bikunin + ITIH1 + ITIH2)
IQR: Interquartile range
ITIH (Hyaluronan-related proteins): Inter-alpha-trypsin inhibitor heavy chains
LYV1 (Hyaluronan-related proteins): Protein Lyl-1
MMTS: Methanethiosulfonate
PαI: Pre-alpha-inhibitor (Bikunin + ITIH3)
PAR: Protease activated receptor
PPBP (hyaluronan-related proteins): Platelet basic protein
SDC: Sodium deoxycholate
SHAP: Serum-derived hyaluronan-associated protein
SIRS: Systemic inflammatory response syndrome
SOFA: Sequential organ failure assessment score
STAB1 (hyaluronan-related proteins): Stabilin-1
TEAB: Triethylammonium bicarbonate
TFA: Trifluoroacetic acid
TGFB1 (Hyaluronan-related proteins): Transforming growth factor beta-1 proprotein
TMT: Tandem mass tag
VCAN (Hyaluronan-related proteins): Versican core protein
