# Supplementary material for: Plasma proteomics in septic shock and alcohol-related pancreatitis: a hyaluronan-centered approach
Source: Clin Proteomics. 2025 Aug 30;22:31. doi: 10.1186/s12014-025-09556-2 (PMC12398169; doi:10.1186/s12014-025-09556-2)
Supplement: Supplementary file 1 — Supplementary Material 1 [file 12014_2025_9556_MOESM1_ESM.docx]

**Supplemental material**

| **Supplemental table S1. Patients characteristics** | | | | | | | | |
| --- | --- | --- | --- | --- | --- | --- | --- | --- |
|  | **Control** | **IQR** | **Sepsis** | **IQR** |  | **Pancreatitis** | **IQR** |  |
|  | **(n=8)** |  | **(n=13)** |  |  | **(n=8)** |  |  |
| Female (%) | 50 |  | 38.5 |  |  | 12.5 |  |  |
| Height (cm) | 175 | 161 - 182 | 175 | 168 - 179 |  | 178 | 171 - 182 |  |
| Weight (kg) | 68 | 55 - 78 | 85 | 72 - 91 | § | 115§ | 82 - 125 | §§ |
| Age (years) | 48 | 38 - 52 | 56 | 48 - 66 |  | 48.5 | 40 - 58 |  |
| SOFA day 1 | N/A |  | 13 | 11 - 15.5 |  | 9 | 5.8 - 9.8 | †† |
| SOFA day 4 | N/A |  | 11 | 7.5 - 14.5 |  | 8 | 3 - 8 | † |
| 28-day mortality (%) | N/A |  | 15.4 |  |  | 0 |  |  |
| Values are presented as median with interquartile range (IQR) or as percentage (%). | | | | | | | | |
| Chi-square test was used for categorical variables | | | | | | | | |
| Mann-Whitney U test post hoc analysis was used after Kruskal-Wallis test for patients with septic shock or pancreatitis compared with controls, § p<0.05, §§ p<0.01 | | | | | | | | |
| Mann-Whitney U test was used to compare SOFA scores between patients with septic shock and pancreatitis for both day 1 and day 4: † p<0.05, †† p<0.01 | | | | | | | | |

| **Supplemental table S2. Clinical parameters and laboratory values** | | | | | | | | | | |
| --- | --- | --- | --- | --- | --- | --- | --- | --- | --- | --- |
|  | **C** | **IQR** | **S1** | **IQR** | **S4** | **IQR** | **P1** | **IQR** | **P4** | **IQR** |
|  | **n=8** |  | **n=13** |  | **n=13** |  | **n=8** |  | **n=8** |  |
| Heart rate (/min) |  |  | **91** | 71 - 110 | **82** | 71 - 89 | **94** | 71 - 108 | **99** | 75 - 110 |
| MAP (mmHg) | **92.5** | 82 - 102 | **73** | 68 - 75 | **80**** | 76 - 92 | **80.5** | 77 - 88 | **93** | 84 - 98 |
| RRsyst (mmHg) | **125.5** | 106 - 141 | **102** | 97 - 114 | **129*** | 115 - 143 | **117** | 109 - 129 | **148*** | 138 - 150 |
| RRdiast (mmHg) | **76** | 71 - 82 | **56** | 52 - 59 | **62** | 55 - 67 | **66** | 59 - 69 | **68** | 61 - 73 |
| CVP (mmHg) |  |  | **14** | 8.5 - 16 | **11** | 9 - 11.8 | **11** | 8.3 - 14.5 |  |  |
| Temperature (℃) | **36.8** | 36.5 - 37.2 | **36.7** | 36.2 - 37.5 | **36.5** | 36.1 - 37.1 | **37.75** | 37.3 - 38.5 | **38.4*** | 37.5 - 39.2 |
| Noradrenaline (mcg/kg/min) |  |  | **0.33** | 0.13 - 0.66 | **0.03*** | 0 - 0.08 | **0.1** | 0.005 - 0.16 | **0** | 0 - 0.05 |
| Lactate (mmol/L) | **0.9** | 0.73 - 1.23 | **2.5** | 2 - 3.45 | **1.3**** | 1.05 - 1.5 | **0.8** | 0.7 - 1.13 | **0.7** | 0.63 - 0.9 |
| Hemoglobin (g/L) | **136.5** | 128 - 140 | **110** | 91 - 115 | **91** | 87 - 108 | **126** | 119 - 141 | **97*** | 84 - 111 |
| Blood leukocyte count (E9/L) | **5.95** | 5.4 - 6.3 | **13.2** | 6.9 - 26.4 | **18.2** | 12.2 - 38.3 | **9.95** | 8.8 - 14.4 | **10.7** | 8.7 - 13 |
| Blood platelet count (E9/L) | **226.5** | 179 - 282 | **63** | 37 - 153 | **53** | 32 - 77 | **137.5** | 116 - 159 | **139.5** | 98 - 170 |
| C: control, S1: septic shock day 1, S4: septic shock day 4, P1: pancreatitis day 1, P4: pancreatitis day 4 | | | | | | | | | | |
| Values as percentage (%) or as median and interquartile range | | | | | | | | | | |
| Wilcoxon test, dependent: day 1 vs day 4, *p <0.05; **p<0.01; ***p<0.001 | | | | | | | | | | |

| **Supplemental table S3. Clinical presentation and blood cultures** | | | |
| --- | --- | --- | --- |
| **Clinical presentation** | **number of patients (n)** | **Blood culture** | **number of patients (n)** |
| Cellulitis | 6 | Negative | 5 |
| Endocarditis | 1 | Pseu. Aeruginosa | 1 |
| Meningitis | 1 | Staph. Aureus | 1 |
| Pneumonia | 3 | Staph. Aureus/Strep. Agalactiae | 1 |
| Pseudomembranous colitis | 2 | Staphylococcus sp. | 1 |
|  |  | Strep. pneumoniae | 4 |

| **Supplemental table S4. Top 10 up- and downregulated proteins in septic shock day 1 vs controls** | | | | | |
| --- | --- | --- | --- | --- | --- |
| **Gene Symbol** | **Gene Name** | **Uniprot ID** | **Log 2 fold change** | **-log10 p-value** | **Adjusted P-value** |
| **Top 10 up-regulated proteins** | | | | | |
| CRP | C-reactive protein | P02741 | 4,4 | 13,76 | 0,00e+00 |
| MMP8 | Matrix metallopeptidase 8 | P22894 | 4,3 | 9,04 | 6,02e-08 |
| CHI3L1 | Chitinase-3-like protein 1 (YKL-40) | P36222 | 4,15 | 8,22 | 2,23e-07 |
| IL1RL1 | Interleukin 1 receptor-like 1 (ST2) | Q01638 | 4,02 | 9,8 | 1,54e-08 |
| COL1A1 | Collagen type I alpha 1 chain | P02452 | 3,75 | 3,45 | 1,92e-03 |
| COL1A2 | Collagen type I alpha 2 chain | P08123 | 3,37 | 3,72 | 1,12e-03 |
| COL2A1 | Collagen type II alpha 1 chain | P02458 | 3,11 | 2,6 | 9,94e-03 |
| SAA2 | Serum amyloid A2 | P0DJI9 | 3,03 | 4,06 | 5,72e-04 |
| SAA1 | Serum amyloid A1 | P0DJI8 | 2,9 | 4,83 | 1,31e-04 |
| REG1B | Regenerating family member 1 beta | P48304 | 2,86 | 5,73 | 2,55e-05 |
| **Top 10 down-regulated proteins** | | | | | |
| SPP2 | Secreted Phosphoprotein 2 | Q13103 | −2,95 | 4,61 | 2,03e-04 |
| TTR | Transthyretin | P02766 | −2,91 | 6,31 | 8,39e-06 |
| SERPINA5 | Serpin family A member 5 | P05154 | −2,60 | 6,4 | 7,27e-06 |
| F12 | Coagulation factor XII | P00748 | −2,46 | 5,97 | 1,56e-05 |
| IGFALS | Insulin like growth factor binding protein acid labile subunit | P35858 | −2,45 | 6,39 | 7,27e-06 |
| IGFBP3 | Insulin like growth factor binding protein 3 | P17936 | −2,31 | 6,77 | 3,42e-06 |
| SERPINA4 | Serpin family A member 4 | P29622 | −2,26 | 12,6 | 0,00e+00 |
| PLG | plasminogen | P00747 | −2,23 | 8,64 | 1,25e-07 |
| GPLD1 | Glycosylphosphatidylinositol specific phospholipase D1 | P80108 | −2,19 | 6,87 | 2,88e-06 |
| HRG | Histidine rich glycoprotein | P04196 | −2,17 | 11,18 | 1,20e-09 |
| Supplement table S4 showing the top-10 upregulated and downregulated proteins in patients with septic shock day 1 compared with controls. The -log 10 p-value is calculated from the post hoc Tukey HSD test. | | | | | |

| **Supplemental table S5. Top 10 up- and downregulated proteins in pancreatitis day 1 vs controls** | | | | | |
| --- | --- | --- | --- | --- | --- |
| **Gene Symbol** | **Gene Name** | **Uniprot ID** | **Log 2 fold change** | **-log10 p-value** | **Adjusted P-value** |
| **Top 10 up-regulated proteins** | | | | | |
| CRP | C-reactive protein | P02741 | 5,09 | 14,09 | 0,00e+00 |
| SAA1 | Serum amyloid A1 | P0DJI8 | 4 | 6,77 | 1,86e-05 |
| S100A8 | S100 calcium binding protein A8 | P05109 | 3,78 | 6,04 | 6,70e-05 |
| S100A12 | S100 calcium binding protein A12 | P80511 | 3,64 | 3,77 | 3,13e-03 |
| CHI3L1 | Chitinase 3 like 1 | P36222 | 3,61 | 5,69 | 1,35e-04 |
| MMP8 | Matrix metallopeptidase 8 | P22894 | 3,31 | 5,24 | 2,52e-04 |
| S100A9 | S100 calcium binding protein A9 | P06702 | 3,2 | 5,22 | 2,52e-04 |
| SAA2 | Serum amyloid A2 | P0DJI9 | 3,02 | 3,35 | 6,37e-03 |
| CPA1 | Carboxypeptidase A1 | P15085 | 3 | 4,02 | 2,42e-03 |
| LBP | Lipopolysaccharide binding protein | P18428 | 2,94 | 5,17 | 2,63e-04 |
| **Top 10 down-regulated proteins** | | | | | |
| SERPINA5 | Serpin family A member 5 | P05154 | −2,15 | 3,91 | 2,82e-03 |
| F12 | Coagulation factor XII | P00748 | −2,02 | 3,56 | 4,60e-03 |
| CNDP1 | Carnosine dipeptidase 1 | Q96KN2 | −1,77 | 3,43 | 5,45e-03 |
| FETUB | Fetuin B | Q9UGM5 | −1,57 | 3,78 | 3,13e-03 |
| HRG | Histidine rich glycoprotein | P04196 | −1,57 | 6,18 | 5,50e-05 |
| HGFAC | HGF activator | Q04756 | −1,53 | 3,81 | 3,08e-03 |
| SERPINA4 | Serpin family A member 4 | P29622 | −1,53 | 6,57 | 2,53e-05 |
| ADA2 | Adenosine deaminase 2 | Q9NZK5 | −1,48 | 2,56 | 2,67e-02 |
| IGFBP3 | Insulin like growth factor binding protein 3 | P17936 | −1,44 | 2,45 | 3,22e-02 |
| GSN | Gelsolin | P06396 | −1,43 | 6,91 | 1,63e-05 |
| Supplement table S5 showing the top-10 upregulated and downregulated proteins in patients with pancreatitis day 1 compared with controls. The -log 10 p-value is calculated from the post hoc Tukey HSD test. | | | | | |

| **Supplemental table S6. Sub-analysis of correlation between ITIH 1-4 and endogenous plasma hyaluronidase inhibition** | | | | |
| --- | --- | --- | --- | --- |
|  | ITIH1 | ITIH2 | ITIH3 | ITIH4 |
| Control | 0.667 | 0.762* | 0.714* | 0.108 |
| Sepsis day 1 | 0.462 | -0.027 | 0.834** | 0.879** |
| Pancreatitis day 1 | 0.071 | 0.167 | 0.143 | -0.333 |
| Sub-analysis of Spearman’s rank-order correlations between log2-fold ratio of relative abundance of ITIH 1-4 and endogenous plasma hyaluronidase inhibition (in %) for controls, patients with septic shock (day 1) and pancreatitis (day 1). | | | | |
| * and **, correlation coefficient is significant at the 0.05 or 0.01 level, respectively. | | | | |

| **Supplemental Table 7. Top 24 enriched GO terms, counts and hyaluronan-related proteins.** | | | |
| --- | --- | --- | --- |
| **Gene Ontology (GO) Term** | **GO aspects** | **Count** | **Hyaluronan-related proteins** |
| Extracellular exosome  GO:0070062 | CC | 10 | CD44, ITIH1, ITIH2, ITIH3, ITIH4, HEXB, AMBP, LYVE1, CLTC, HEXA |
| Extracellular region  GO:0005576 | CC | 10 | ITIH1, ITIH2, ITIH3, ITIH4, TGFB1, HABP2, PPBP, HEXB, VCAN, AMBP |
| Plasma membrane  GO:0005886 | CC | 7 | CD44, ITIH4, TGFB1, AMBP, LYVE1, CLTC, STAB1 |
| Hyaluronic acid binding  GO:0005540 | MF | 6 | CD44, ITIH1, ITIH2, VCAN, LYVE1, STAB1 |
| Collagen-containing extracellular matrix  GO:0062023 | CC | 6 | ITIH1, ITIH2, ITIH4, TGFB1, VCAN, AMBP |
| Hyaluronan catabolic process  GO:0030214 | BP | 5 | CD44, TGFB1, HEXB, LYVE1, HEXA |
| Serine-type endopeptidase inhibitor activity  GO:0004867 | MF | 5 | ITIH1, ITIH2, ITIH3, ITIH4, AMBP |
| Negative regulation of peptidase activity  GO:0010466 | BP | 5 | ITIH1, ITIH2, ITIH3, ITIH4, AMBP |
| Blood microparticle  GO:0072562 | CC | 5 | ITIH1, ITIH2, ITIH4, TGFB1, AMBP |
| Extracellular space  GO:0005615 | CC | 5 | TGFB1, HABP2, PPBP, VCAN, AMBP |
| Cell adhesion  GO:0007155 | BP | 5 | CD44, HABP2, VCAN, AMBP, STAB1 |
| Membrane  GO:0016020 | CC | 5 | HEXB, VCAN, LYVE1, CLTC, HEXA |
| Hyaluronan metabolic process  GO:0030212 | BP | 4 | ITIH1, ITIH2, ITIH3, ITIH4 |
| Calcium ion binding  GO:0005509 | MF | 4 | ITIH1, HABP2, VCAN, STAB1 |
| Cytosol  GO:0005829 | CC | 4 | CD44, AMBP, CLTC, HEXA |
| Receptor-mediated endocytosis  GO:0006898 | BP | 3 | LYVE1, CLTC, STAB1 |
| Cell surface  GO:0009986 | CC | 3 | CD44, 'TGFB1, AMBP |
| Lysosome  GO:0005764 | CC | 3 | HEXB, CLTC, 'HEXA |
| Skeletal system development  GO:0001501 | BP | 3 | HEXB, VCAN, HEXA |
| Carbohydrate binding  GO:0030246 | MF | 3 | ITIH1, VCAN, AMBP |
| GO aspects: Molecular Function (MF), Cellular Component (CC), and Biological Process (BP). | | | |
